# Brief self-affirmation intervention for adults with psoriasis for reducing anxiety and depression and boosting well-being: Evidence from a randomized controlled trial

**DOI:** 10.1017/S0033291721004499

**Published:** 2023-04

**Authors:** Patryk Łakuta

**Affiliations:** Institute of Psychology, SWPS University of Social Sciences and Humanities, Warsaw, Poland and Institute of Psychology, Cardinal Stefan Wyszyński University, Warsaw, Poland

**Keywords:** anxiety, depression, implementation intentions, mental health, psoriasis, randomized controlled trial, self-affirmation

## Abstract

**Background:**

There are relatively few studies to address mental health implications of self-affirming, especially across groups experiencing a chronic health condition. In this study, short- and longer-term effects of a brief self-affirmation intervention framed in terms of implementation intentions (if-then plans with self-affirming cognitions; S-AII) were evaluated against an active control group (non-affirming implementation intentions; N-AII), matched to the target condition, and mere goal intention condition (a non-active control) in adults with psoriasis. The three pre-registered primary outcomes captured depression, anxiety, and well-being.

**Methods:**

Adults with psoriasis (*N* = 175; *M*_age_ = 36.53, s.d. = 11.52) were randomized into S-AII, N-AII, or control. Participants' mental health outcomes were assessed prior to randomization (at baseline), at week 2 (post-intervention), and at a 1-month follow-up.

**Results:**

Linear mixed models were used and results were reported on the intention-to-treat principle. Analyses revealed that S-AII exerted significantly more improvement in the course of well-being (*d*s > 0.25), depressive symptoms (*d*s > −0.40), and anxiety (*d*s > −0.45) than the N-AII and control group at 2-week post-intervention. Though the differences between groups faded at 1-month follow-up, the within-group changes over time for S-AII in all mental health outcomes remained significant.

**Conclusions:**

Brief and low-intensity S-AII intervention exerted in the short-term a considerable impact on mental health outcomes. The S-AII shows promising results as a relevant public mental health strategy for enhancing well-being and reducing psychological distress. Future studies could consider whether these effects can be further enhanced with booster interventions.

Building from growing literature on beneficial effects of self-affirmation interventions (Cohen & Sherman, 2014; Dutcher et al., 2020; Howell, 2017; Sherman, 2013), this study tested whether encouraging to cultivate a sense of self as worthy, adequate, and efficacious can provide improvements on mental health indicators. The current research sought to address gaps in the literature on the applicability of self-affirmation theory and mental health implications of self-affirming among truly at-risk populations, with the burden of chronic disease, employing a robust RCT design with an active-control group and longer-term follow-up assessment. To further our understanding of whether and how self-affirming can be leveraged to induce better mental health outcomes, the present study directly compares the effects of self-affirmation on both a generation of positive effects (i.e. on boosting well-being) as net benefits on psychological functioning and a reduction of negative mental health outcomes – depression and anxiety – for adults with psoriasis.

Psoriasis is a global problem with at least 100 million individuals affected worldwide, most commonly in populations of northern Europe (World Health Organization [WHO], 2016). Analysis of the position of psoriasis patients in society implies that they suffer from a pervasive and chronic societal threat of stigmatization from a visible skin condition, that is often seen as contagious (Chen, Beck, Tan, & Koo, 2018; Donigan, Pascoe, & Kimball, 2015; Rasmussen, Kragballe, Maindal, & Lomborg, 2018), ipso facto, contributing to detrimental effects on their mental health. There is a sound evidence base demonstrating a high prevalence of anxiety and depression in psoriasis patients (e.g. Dalgard et al., 2015; Fleming et al., 2017; Kleyn et al., 2020; Koo et al., 2017). Research on psychosocial interventions addressing psychosocial burden in adults with psoriasis is, however, very limited; though being a matter of prominent importance (Kleyn et al., 2020; Koo et al., 2017; White et al., 2020; Zill et al., 2019).

Previous research considering a high prevalence of anxiety and depression in psoriasis has underscored, besides issues around its social stigmatization, the importance of addressing self-esteem and self-image impairments (e.g. Łakuta, Marcinkiewicz, Bergler-Czop, & Brzezińska-Wcisło, 2016; Słomian, Łakuta, Bergler-Czop, & Brzezińska-Wcisło, 2018). It has also been shown that there is a group of patients who are better able to deal with the psychological consequences of psoriasis than others (e.g. Rzeszutek, Podkowa, Pięta, Pankowski, & Cyran-Stemplewska, 2020). Regarding what makes a difference, it was pointed that a positive body image is not enough to preserve a high level of life satisfaction when an individual does not recognize the availability of his/her resources (e.g. vital, spiritual, or family resources). And, here is what self-affirmation can offer. According to the perspective model of self-affirmation effects (Critcher & Dunning, 2015), whereas self-threats can constrict the self-concept to focus on threatened aspects, affirmations restore a broader perspective on the self; which as a result blunting the impact of a constricted self that was disproportionately influenced by the threat, thereby permitting a person to draw on its broader dispositional resources. In a series of elegant experiments, Critcher and Dunning (2015) gave support to the notion that affirming self does not result in inflated self-worth, but expands self-concept in battle by recognizing additional identities in the self (e.g. resourceful, efficacious, honest, a good partner/son/daughter, etc.). It leads an individual to see how a certain threat is situated within the broader field of the self – the threat becomes restricted to a narrowed part.

However, if such positive self-talk is not one's natural tendency (c.f. Brady et al., 2016; Emanuel et al., 2018), forming a new habit must take time and effort. Recognizing that, and given the pervasiveness and range of self-evaluative threats in psoriasis, this study employed the intervention framed in terms of implementation intentions (IIs) – if-then planning – constituting an effective way of self-regulatory goals attainment and building new habits (Gollwitzer, 2014). The self-affirmation intervention tested in the current study, in which an individual forms an if-then plan with self-affirming cognitions (self-affirming implementation intention – S-AII), though already well-rooted within self-affirmation research (e.g. Armitage, 2016; Armitage, Harris, & Arden, 2011; Łakuta, 2020a; Morgan & Atkin, 2016; Morgan & Harris, 2015), differs by design from typical self-affirmation writing exercises (see McQueen & Klein, 2006). Writing tasks require verbal fluency, and their demanding and time-consuming nature makes the type of intervention less desirable (c.f. Armitage et al., 2011; Napper, Harris, & Epton, 2009). In contrast, the S-AII provides a brief, simple, and efficient way to self-affirm, with greater standardization of the intervention and a greater ‘control equivalence’ in experimental self-affirmation research. The S-AII brings also many other advantages that put it in a more favorable position.

The intervention adopted in this study also reflects the three contextual factors – the presence of threat (i.e. there must be a threat to self-adequacy or self-integrity), the availability of resources (i.e. there must exist some infrastructure or other instrumental content), and the timeliness of the self-affirmation (i.e. it must be delivered in temporal proximity to a psychological threat) – to be fulfilled to enable significant and positive self-affirmation effects specified under theoretical assumptions of the Trigger and Channel Framework (Ferrer & Cohen, 2019). Given the strategic automaticity produced by IIs, also in regard to emotion regulation processes (Martiny-Huenger, Martiny, Parks-Stamm, Pfeiffer, & Gollwitzer, 2017; Wieber, Thürmer, & Gollwitzer, 2015; see also Hallam et al., 2015; Huang, Chen, Gao, Yang, & Yuan, 2020; Webb, Schweiger Gallo, Miles, Gollwitzer, & Sheeran, 2012), the if-then structure makes self-affirming cognitions accessible once the self-system is threatened. Forming IIs results in activation primarily areas associated with bottom-up, automatic action control (stimulus-controlled action); differently than goal striving with mere goal intentions that recruit regions associated with top-down processing and deliberate action control (Bieleke, Keller, & Gollwitzer, 2021). Created by IIs associative links between a critical situation (*if*-part) and a goal-directed response (*then*-part) not only automatically facilitate the initiation of the specified response once this situation is encountered (e.g. down-regulation of negative emotions), but also are stable over time, and the effect can generalize to similar situations, still triggering the planned response; thus allow having prepared planned actions for future goal-relevant situations (Huang et al., 2020; for a review, see Bieleke et al., 2021). The processes regarding bottom-up – stimulus-controlled action and the potential of generalization, in particular – might be relevant to contribute to the improvement of emotional regulation especially among people struggling with multiple emotional stressors.

## The present study

In this study, short- and longer-term effects of a brief II-based self-affirmation intervention (if-then plans with self-affirming cognitions; S-AII) were evaluated against an active control group with distraction strategies (non-affirming implementation intentions; N-AII), and mere goal intention condition (a non-active control). The active control condition was chosen to allow the effect of the S-AII to be examined above and beyond the effect of setting (non-self-affirming) IIs. The passive control condition was chosen as the basic comparator to allow the effect of the S-AII to be examined above and beyond the effect of simply setting goal intention. Three pre-registered primary outcomes captured depression, anxiety, and well-being. For the first time, adopting RCT design, it was tested whether S-AII intervention for adults with psoriasis can exert beneficial effects both at the primary endpoint, that was set to week 2 (post-intervention), and at follow-up, 1 month after, providing a test of the durability of any observed effects. In the study, eight pre-registered hypotheses were tested (see online Supplementary Material).

Based on prior evidence drawn from other populations (Cohen & Sherman, 2014; Howell, 2017), it was expected that S-AII would generate effects both in terms of reduction in negative mental health outcomes (i.e. depression and anxiety) and net benefits for overall well-being. In this study, in line with many theoretical considerations (e.g. Keyes, 2005; Seligman, 2011; Wong, 2011), it was adopted the conceptualization of well-being as involving both hedonic and eudaimonic aspects. From a theoretical standpoint, because self-affirmation prompts people to reflect on their values, strengths, and/or most important relationships, it may also encourage them to engage in activities that are congruent with those values^†^^1^ – activities that are happiness-enhancing (hedonic well-being) and/or reinforce vital aspects of eudaimonic well-being as embracing positive relations with others, fulfillment of psychological needs, and experience of meaning and purpose in life (Keyes, 1998, 2005; Ryan & Deci, 2001; Ryff, 1989). To date, the small number of studies that directly investigated the effects on well-being has resulted in promising but mixed findings (Howell, 2017). Moreover, with notable exceptions, most affirmation interventions have been tested in student or non-clinical samples, with experimental manipulations of threat, rather than truly at-risk populations experiencing chronic and acute stressors. Relatedly, self-affirmation interventions require further research to support or refute their status as a well-being intervention.

Besides primary outcomes, the study tested secondary effects of the intervention in terms of positive prosocial- and self-directed feelings, and emotional attitude toward the body. Moreover, given that under threat, the size of the working self-concept is constricted and that the effect can be undermined through self-affirmation that restores a broader view on the self, enabling to draw on extensive dispositional resources and to promote defocusing and adopting broader perspective (Critcher & Dunning, 2015); it seems valuable to examine potential cognitive processes that may reflect those effects. This study evaluated whether self-affirmations alter thought (cognitive) processes in terms of cognitive emotion regulation strategies, e.g. putting into perspective or catastrophizing (c.f. Garnefski, Kraaij, & Spinhoven, 2002). Notably, in light of evidence that the maladaptive strategies (e.g. self-blame, rumination, catastrophizing) have been shown to be strongly and consistently related to psychopathology, especially affective disorders (Aldao, Nolen-Hoeksema, & Schweizer, 2010), the potential of altering these cognitive processes through self-affirmation could be of crucial importance for achieving successful long-term outcomes.

## Method

### Participants and procedure

**Fig. 1.**
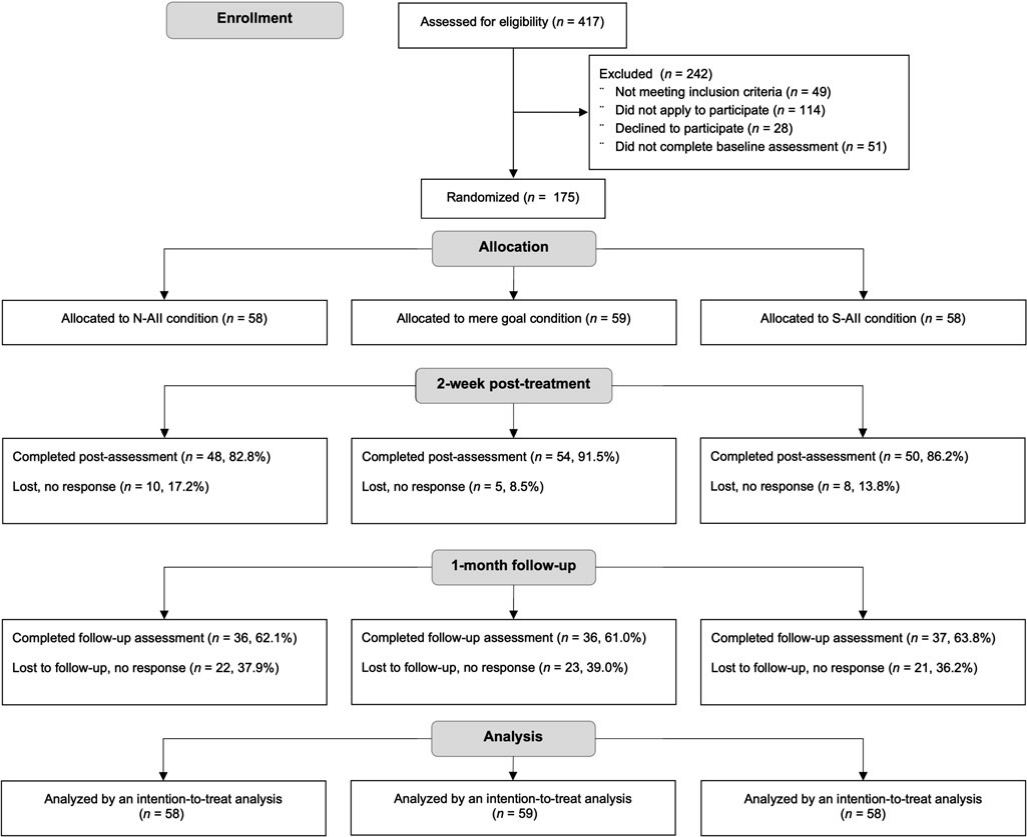
CONSORT flowchart of enrolment, intervention allocation, follow-up, attrition, and data analysis.

The prospective, three-arm RCT with parallel group design hosted on the Qualtrics online research platform was conducted between 2019 and 2021 with ethical approval granted by the University's Institutional Review Board. The three arms of the study were as follows: S-AII condition, N-AII condition, and mere goal intention condition. Participants were evaluated prior to randomization (baseline), at week 2 (post-intervention), and at the final point of the study (1-month following the post-intervention assessment). The study was pre-registered on the Open Science Framework before any data were collected (available at https://osf.io/w8gfn). All individuals prior to participation in the study completed informed consent forms. Participant recruitment, randomization, and progress through the study are presented in the CONSORT flowchart in Fig. 1.

Participants were recruited through an online advertisement (social media, e.g. Facebook) as well as a series of offline methods (i.e. flyers and posters in psoriasis patient associations, hospitals, and outpatient clinics); and screened before entering the study. Individuals were eligible for participation if they: were 18 years or older; had physician-diagnosed psoriasis; had Internet access and provided a valid e-mail address; and read and accepted the informed consent. Exclusion criteria were the following: participating in other psychosocial or pharmacological treatments or being enrolled in a trial or in any research on mental health (e.g. a clinical trial of an investigational medical product). A total of 417 psoriasis patients were screened for eligibility, of whom 175 completed the baseline assessment and were randomized to either S-AII, N-AII, or mere goal condition. The study information sheet informed participants that they would be asked (besides to complete questionnaires on mood, the disease, and coping with psoriasis) to complete a written task concerning their functioning in the following weeks, without specifying conditions, thus participants were unaware of the group assignment. As randomization to groups was conducted automatically, the research team was blinded to allocation. Participants were randomized on a 1:1:1 ratio without any constraints through the Qualtrics randomizer feature. A system automatically provided the participant's unique identification code in a password-protected online research platform.

Of the individuals in the randomized sample, the majority were female (69.1%), married/cohabited with a partner (68.6%), had paid employment (65.2%), and were highly educated (43.4%). Participants were aged 18–71 years (*M* = 36.53 years, s.d. = 11.52) with the mean psoriasis severity of 10.45 SAPASI score (s.d. = 8.01, range: 0–48; 45.7% with moderate or severe psoriasis, i.e. score ⩾10; and 2.9% of participants were in remission). Plaque psoriasis made up 85.1% of cases in the sample. Under biological treatment were only 8.0% of participants.

A minimum sample size of 168 participants (56 per group) was estimated by *a priori* power analysis for detecting a medium effect (*f* = 0.25) of S-AII (c.f. Morgan & Atkin, 2016; Morgan & Harris, 2015; see also Gollwitzer & Sheeran, 2006; Webb et al., 2012) in a mixed-model analysis of variance with three groups and three points of measurement (i.e. baseline to 1-month follow-up), with power set to 95%, a significance level of 5%, and a correlation of 0.5 between measures. To minimize the dropout of the study, a monetary incentive of about $25 was given to participants who completed all measurements.

### Intervention and control condition materials

#### Self-affirming implementation intention condition

The S-AII is a brief standardized self-affirmation intervention in which participants are asked to form an if-then plan with self-affirming cognitions, for example, ‘If I feel sad, threatened or anxious, then I will think about the things I value about myself’ (c.f. Armitage et al., 2011; see also Morgan & Atkin, 2016; Morgan & Harris, 2015). Thus, the task employs a self-affirm paradigm but also makes use of the if-then structure of IIs (Gollwitzer, 2014). Participants were provided with an II prompt in the form of a sentence stem: ‘If I feel sad, threatened or anxious, then I will…’, where ‘feeling sad, threatened or anxious’ is the critical situation; and a choice of (four) suitable self-affirming responses, with which to complete the sentence, included, e.g. ‘…think about things that are important to me’. Participants completed the task by ticking a box with one preferred self-affirmation and writing down the full plan. Afterward, participants were asked to read the plan three times and to repeat it silently to themselves.

#### Non-affirming implementation intention condition (active control)

Participants in the active control group were received instruction to formulate an if-then plan with no opportunity to self-affirm, that is N-AII. Participants in the N-AII condition were given the same sentence stem as those in the S-AII condition; however, the four options that followed were designed based on distraction strategies to ensure that there is no opportunity for participants to self-affirm, e.g. ‘…think about the shops and buildings I pass on a journey I travel regularly’ (c.f. Morgan & Atkin, 2016; Morgan & Harris, 2015). Participants completed the task by ticking a box with one preferred distraction strategy and writing down the full plan. Finally, they were instructed to read the plan three times and to repeat it silently to themselves.

#### Mere goal intention condition (passive control)

Participants in the non-active control group were received instruction to merely identifying and forming a goal intention regarding adaptive functioning and feeling better in the next weeks (i.e. an intention in the format ‘I want to achieve X/perform behavior X!’, with X representing desired future, outcome, or behavior) (c.f. Gollwitzer, 2014). Participants were asked to write out the goal intention they set. Finally, they were instructed to read the goal three times and to repeat it silently to themselves.

### Measures

Besides socio-demographic characteristics (i.e. age, gender, marital status, educational level), the disease severity based on the Self-Administered Psoriasis Area and Severity Index (SAPASI; Feldman et al., 1996; Sampogna et al., 2003), primary and secondary outcome measures were administered at baseline, post-intervention, and 1-month follow-up (for a detailed description, see online Supplementary Materials).

#### Primary outcomes

Depression severity was measured by the nine-item Patient Health Questionnaire (PHQ-9; Kroenke, Spitzer, & Williams, 2001). Anxiety severity was measured by the seven-item Generalized Anxiety Disorder Scale (GAD-7; Spitzer, Kroenke, Williams, & Löwe, 2006). Well-being was measured with the 14-item Mental Health Continuum-Short Form (MHC-SF; Karaś, Cieciuch, & Keyes, 2014; Keyes, 2002; Żemojtel-Piotrowska et al., 2018).

#### Secondary outcomes

Cognitive emotion regulation strategies were measured by the Cognitive Emotion Regulation Questionnaire (Garnefski et al., 2002). Positive other- and self-directed feelings were measured by asking participants to indicate how often they have experienced five prosocial (e.g. love, empathic, connected, grateful) and positive feelings directed toward themselves (e.g. pride, feeling strong, in control) in their daily lives, respectively (Crocker, Niiya, & Mischkowski, 2008; Thomaes, Bushman, de Castro, & Reijntjes, 2012). Negative emotional attitude toward the body was measured by the nine-item Body Emotions Scale (Sakson-Obada, 2009).

### Statistical analyses

Following the intention-to-treat principle, main analyses were conducted using linear mixed models (LMMs) to assess differences between groups at post-intervention and at 1-month follow-up assessments in the primary and secondary outcome variables. LMM is a highly flexible approach that makes use of all data, that each participant provided, in parameter estimation and significance testing. Analyses were thus performed using the data of all randomized participants, including participants with missing data, minimizing power loss (Sullivan, White, Salter, Ryan, & Lee, 2018). For each outcome measure, with baseline, post-intervention, and follow-up as three time-points, a maximum likelihood was used as the method of estimation with an autoregressive (heterogeneous) covariance structure [ARH(1)] type, which assumes that measurements that are closer in time are more strongly correlated than those that are further apart. The group assignment, time point, and the interaction between group and time were included in the models as fixed effects. Where LMMs demonstrated a significant group × time interaction, post hoc pairwise comparisons of means estimated from the models were performed. The magnitude of the treatment effect within and between the groups was assessed using Cohen's *d* statistic^2^.

In addition, based on a completer analysis, intervention effects regarding main outcomes are reported using three methods to classify change in terms of rates of individuals demonstrating: (1) clinically significant depressive/anxiety symptoms (based on widely used cut-off points of ⩾10 with the optimal balance between sensitivity, specificity, and positive predictive value on the PHQ-9 and GAD-7; see Kroenke, Spitzer, Williams, & Löwe, 2010; Mitchell, Yadegarfar, Gill, & Stubbs, 2016; Plummer, Manea, Trepel, & McMillan, 2016); (2) a reliable change index (RCI) for sum scores between measurement points – change classified, with 95% confidence, greater than expected from measurement error (Jacobson & Truax, 1991); and (3) a minimal clinically important difference (MCID; Mouelhi, Jouve, Castelli, & Gentile, 2020) for sum scores between measurement points – smallest change in a treatment outcome that can be considered to be clinically important (for more detailed information, see online Supplementary Material). To compare the proportion of participants based on the given criterion across conditions, χ^2^ tests were performed.

## Results

### Randomization check and attrition analysis

A series of ANOVA and χ^2^ tests indicated that the three study arms did not significantly differ regarding sociodemographic characteristics, the disease severity, or outcome measures at baseline (all *p*s > 0.152), indicating successful randomization (see Table 1).

**Table 1.** Baseline characteristics of the participants (*N* = 175)

	Control (*n* = 59)	N-AII (*n* = 58)	S-AII (*n* = 58)	Test statistics
*Demographics*				
Age (years), *M* (s.d.)	36.56 (11.56)	35.55 (11.28)	37.48 (11.84)	*F*_(__2, 172)_ = 0.41, *p* = 0.668
Gender, *n* (%)				χ^2^(2, *N* = 175) = 0.01, *p* = 0.997
Female	41 (69.5%)	40 (69.0%)	40 (69.0%)	
Male	18 (30.5%)	18 (31.0%)	18 (31.0%)	
Marital status, *n* (%)				χ^2^(2, *N* = 175) = 0.40, *p* = 0.821
Married/cohabiting	41 (69.5%)	41 (70.7%)	38 (65.5%)	
Not married (single, divorced, widowed)	18 (30.5%)	17 (29.3%)	20 (34.5%)	
Education level (highest level completed), *n* (%)				χ^2^(4, *N* = 175) = 0.29, *p* = 0.990
Low (primary school, vocational education)	11 (18.6%)	12 (20.7%)	12 (20.7%)	
Intermediate (upper secondary education)	23 (39.0%)	20 (34.5%)	21 (36.2%)	
High (tertiary education, university degree)	25 (42.4%)	26 (44.8%)	25 (43.1%)	
Work status, *n* (%)				χ^2^(6, *N* = 175) = 3.19, *p* = 0.784
Student	5 (8.5%)	8 (13.8%)	5 (8.6%)	
Paid employment	37 (62.7%)	37 (63.8%)	40 (69.0%)	
Unemployed	8 (13.6%)	5 (8.6%)	8 (13.8%)	
Pensioner or retired	9 (15.2%)	8 (13.8%)	5 (8.6%)	
Psoriasis severity (SAPASI), *M* (s.d.)	10.99 (6.67)	11.11 (8.80)	9.23 (8.42)	*F*_(__2, 172)_ = 1.01, *p* = 0.367
*Primary outcomes*				
PHQ-9, *M* (S.D.)	9.83 (6.12)	11.40 (6.54)	10.28 (6.07)	*F*_(__2, 172)_ = 0.97, *p* = 0.380
Prevalence of depression (PHQ-9 ⩾ 10), *n* (%)	27 (45.8%)	32 (55.2%)	28 (48.3%)	χ^2^(2, *N* = 175) = 1.11, *p* = 0.575
GAD-7, *M* (S.D.)	9.59 (5.24)	10.02 (5.39)	9.22 (4.98)	*F*_(__2, 172)_ = 0.34, *p* = 0.714
Prevalence of anxiety (GAD-7 ⩾ 10), *n* (%)	28 (47.5%)	30 (51.7%)	22 (38.0%)	χ^2^(2, *N* = 175) = 2.33, *p* = 0.312
MHC-SF (total score), *M* (S.D.)	27.31 (15.50)	24.45 (16.36)	26.40 (14.66)	*F*_(__2, 172)_ = 0.52, *p* = 0.598
MHC-SF EW, *M* (S.D.)	6.19 (3.89)	5.57 (4.07)	5.79 (3.18)	*F*_(__2, 172)_ = 0.41, *p* = 0.664
MHC-SF SW, *M* (S.D.)	6.95 (5.37)	6.98 (5.93)	6.71 (5.81)	*F*_(__2, 172)_ = 0.04, *p* = 0.960
MHC-SF PW, *M* (S.D.)	14.17 (7.58)	11.90 (7.57)	13.90 (7.37)	*F*_(__2, 172)_ = 1.59, *p* = 0.207

GAD-7, seven-item Generalized Anxiety Disorder Scale; MHC-SF, Mental Health Continuum-Short Form; EW, emotional well-being subscale; SW, social well-being subscale; PW, psychological well-being subscale; PHQ-9, nine-item Patient Health Questionnaire; SAPASI, Self-Administered Psoriasis Area and Severity Index (the range of absolute SAPASI scores is 0–72); N-AII, non-affirming implementation intention condition; S-AII, self-affirming implementation intention condition.

At post-intervention and 1-month follow-up, data were available for 152 and 109 participants, respectively (see Fig. 1). Attrition between randomization and completion of the post-intervention and follow-up assessments was found to not differ by condition (all *p*s > 0.368), indicating that the dropout was non-systematic.

### Intervention effects on primary outcomes

An overview of the results of LMMs is shown in Table 2. The estimated mean differences and 95% confidence intervals as generated by LMMs are presented in Table 3 (estimated means are presented in online Supplementary Table S2).

**Table 2.** LMM analyses on the primary and secondary outcomes (*N* = 175)

	Time	Condition	Time × condition
*Primary outcomes*			
PHQ-9	*F*_(__2, 215)_ = 4.73, *p* = 0.010	*F*_(__2, 173)_ = 2.03, *p* = 0.134	*F*_(__4, 215)_ = 3.76, *p* = 0.006
GAD-7	*F*_(__2, 201)_ = 10.58, *p* < 0.001	*F*_(__2, 175)_ = 2.16, *p* = 0.118	*F*_(__4, 201)_ = 3.25, *p* = 0.013
MHC-SF (total score)	*F*_(__2, 211)_ = 1.90, *p* = 0.152	*F*_(__2, 177)_ = 1.31, *p* = 0.272	*F*_(__4, 211)_ = 2.56, *p* = 0.040
MHC-SF EW^a^	*F*_(__2, 207)_ = 6.14, *p* = 0.003	*F*_(__2, 176)_ = 0.56, *p* = 0.572	*F*_(__4, 207)_ = 0.79, *p* = 0.533
MHC-SF SW^a^	*F*_(__2, 209)_ = 2.93, *p* = 0.056	*F*_(__2, 178)_ = 0.59, *p* = 0.554	*F*_(__4, 209)_ = 3.51, *p* = 0.009
MHC-SF PW^a^	*F*_(__2, 205)_ = 0.45, *p* = 0.637	*F*_(__2, 176)_ = 2.09, *p* = 0.127	*F*_(__4, 205)_ = 1.54, *p* = 0.192
*Secondary outcomes*			
Refocusing on planning	*F*_(__2, 181)_ = 2.11, *p* = 0.124	*F*_(__2, 161)_ = 2.37, *p* = 0.096	*F*_(__4, 181)_ = 1.36, *p* = 0.248
Positive reappraisal	*F*_(__2, 197)_ = 0.68, *p* = 0.509	*F*_(__2, 170)_ = 1.21, *p* = 0.300	*F*_(__4, 197)_ = 0.99, *p* = 0.410
Positive refocusing	*F*_(__2, 187)_ = 6.57, *p* = 0.002	*F*_(__2, 174)_ = 0.76, *p* = 0.469	*F*_(__4, 187)_ = 1.83, *p* = 0.125
Putting into perspective	*F*_(__2, 184)_ = 0.08, *p* = 0.926	*F*_(__2, 178)_ = 2.46, *p* = 0.088	*F*_(__4, 184)_ = 1.00, *p* = 0.411
Acceptance	*F*_(__2, 174)_ = 0.30, *p* = 0.741	*F*_(__2, 174)_ = 0.57, *p* = 0.568	*F*_(__4, 174)_ = 0.73, *p* = 0.574
Self-blame	*F*_(__2, 181)_ = 5.14, *p* = 0.007	*F*_(__2, 169)_ = 0.56, *p* = 0.571	*F*_(__4, 181)_ = 0.34, *p* = 0.848
Rumination	*F*_(__2, 184)_ = 3.74, *p* = 0.026	*F*_(__2, 167)_ = 0.40, *p* = 0.672	*F*_(__4, 184)_ = 0.74, *p* = 0.564
Catastrophizing	*F*_(__2, 184)_ = 3.78, *p* = 0.025	*F*_(__2, 170)_ = 2.59, *p* = 0.078	*F*_(__4, 184)_ = 2.50, *p* = 0.044
Other-blame	*F*_(__2, 181)_ = 3.12, *p* = 0.047	*F*_(__2, 162)_ = 0.29, *p* = 0.751	*F*_(__4, 181)_ = 0.12, *p* = 0.977
Positive other-directed feelings	*F*_(__2, 267)_ = 2.99, *p* = 0.052	*F*_(__2, 178)_ = 0.36, *p* = 0.699	*F*_(__4, 267)_ = 2.18, *p* = 0.071
Positive self-directed feelings	*F*_(__2, 270)_ = 1.79, *p* = 0.169	*F*_(__2, 179)_ = 0.37, *p* = 0.691	*F*_(__4, 270)_ = 0.85, *p* = 0.496
Emotional attitude toward the body	*F*_(__2, 191)_ = 5.46, *p* = 0.005	*F*_(__2, 181)_ = 1.49, *p* = 0.228	*F*_(__4, 191)_ = 1.14, *p* = 0.340

Results are reported on an intention-to-treat basis. Linear mixed models were used with Satterthwaite method for degrees of freedom. GAD-7, seven-item Generalized Anxiety Disorder Scale; MHC-SF, Mental Health Continuum-Short Form; EW, emotional well-being subscale; SW, social well-being subscale; PW, psychological well-being subscale; PHQ-9, nine-item Patient Health Questionnaire.

a Analyses conducted for exploratory purposes.

**Table 3.** Estimated mean differences between groups at post-intervention and at 1-month follow-up assessments with standardized between-group effect sizes (*N* = 175)

	Condition	Estimated mean difference (95% CI)	*p*	Cohen's *d*
*Post-treatment*				
PHQ-9	N-AII *v*. control	1.07 (−1.16; 3.31)	0.343	0.17
	S-AII *v*. Control	−2.41 (−4.63; −0.19)	0.034	−0.40
	S-AII *v*. N-AII	−3.48 (−5.74; −1.23)	0.003	−0.55
GAD-7	N-AII *v*. control	−0.73 (−2.58; 1.11)	0.434	−0.14
	S-AII *v*. control	−3.05 (−4.89; −1.22)	0.001	−0.60
	S-AII *v*. N-AII	−2.32 (−4.19; −0.45)	0.015	−0.45
MHC-SF (total score)	N-AII *v*. control	−2.58 (−8.15; 2.99)	0.361	−0.16
	S-AII *v*. control	3.81 (−1.74; 9.36)	0.177	0.25
	S-AII *v*. N-AII	6.39 (0.77–12.01)	0.026	0.41
*1-month follow-up*				
PHQ-9	N-AII *v*. control	0.24 (−2.20; 2.67)	0.849	0.04
	S-AII *v*. Control	−1.24 (−3.66; 1.18)	0.312	−0.20
	S-AII *v*. N-AII	−1.47 (−3.91; 0.96)	0.233	−0.23
GAD-7	N-AII *v*. control	−0.50 (−2.73; 1.73)	0.657	−0.09
	S-AII *v*. control	−1.39 (−3.61; 0.82)	0.216	−0.27
	S-AII *v*. N-AII	−0.89 (−3.12; 1.34)	0.432	−0.17
MHC-SF (total score)	N-AII *v*. control	−3.12 (−9.83; 3.58)	0.359	−0.20
	S-AII *v*. control	1.79 (−4.89; 8.46)	0.597	0.12
	S-AII *v*. N-AII	4.91 (−1.81; 11.63)	0.151	0.32

GAD-7, seven-item Generalized Anxiety Disorder Scale; MHC-SF, Mental Health Continuum-Short Form; PHQ-9, nine-item Patient Health Questionnaire.

With regard to the main outcomes, examining the differences in changes over time between the study conditions, significant condition × time interactions were found (all *p*s < 0.05), suggesting that the changes in the main outcomes over time differed per condition. In line with post hoc LMM analyses, the S-AII group, as opposed to the N-AII and control group, showed meaningful improvements in well-being (Cohen's *d* > 0.25) and a significant reduction in depression and anxiety symptoms at post-intervention (Cohen's *d*s from −0.40 to −0.60). At the 1-month follow-up assessment, however, there were no significant differences between the conditions, and in most cases, between-group effect sizes were small to negligible (see Table 3).

Based on the within-group estimates (see online Supplementary Table S2), the S-AII intervention yielded small to medium effect sizes at post-intervention on well-being (*d* = 0.25), anxiety (*d* = −0.55), and depression (*d* = −0.44). The improvement was sustained during the 1-month follow-up period. The results showed also some parallel improvements in depression and anxiety symptoms over time within the N-AII group (*d*s from −0.22 to −0.44).

Additionally, exploratory analyses were undertaken to describe differences in terms of three dimensions of well-being (Table 2). LMMs showed a significant group × time interaction only on the social dimension of well-being. At post-intervention, a significant change over time on social well-being was observed only within the S-AII condition (*M*_diff_ = 1.63, 95% Cl 0.61–2.66; *p* = 0.002; *d* = 0.28). The improvement was sustained during 1-month follow-up (*M*_diff_ = 1.84, 95% Cl 0.27–3.41; *p* = 0.022; *d* = 0.32).

### Intervention effects on secondary outcomes

LMMs demonstrated mostly no statistically significant group × time interaction effects (Table 2), indicating similar levels over time of cognitive emotion regulation strategies, positive other-directed and self-directed feelings, and emotional attitude toward the body in the three study arms. Changes over time from baseline to 1-month follow-up (all *p*s < 0.05) were detected in terms of an increase in positive refocusing and reduction in self-blame, rumination, other-blame, and negative emotional attitude toward the body within all the study groups. Effect sizes were small to negligible.

There was one exception. LMM showed a significant group × time interaction on one of the coping strategies – catastrophizing (see Table 2). Significant group differences with medium effect sizes were seen between the S-AII and control group (*M*_diff_ = −1.72, 95% Cl −3.00 to −0.43; *p* = 0.009; *d* = −0.51) and S-AII and N-AII (*M*_diff_ = −1.82, 95% Cl −3.13 to −0.51; *p* = 0.007; *d* = −0.58) at the post-intervention assessment. Moreover, only within the S-AII was observed a significant change post-intervention (*M*_diff_ = −1.31, 95% Cl −2.03 to −0.58; *p* < 0.001; *d* = −0.42). The improvement – the decreased use of catastrophizing – was sustained during 1-month follow-up (*M*_diff_ = −1.28, 95% Cl −2.39 to −0.18; *p* = 0.023; *d* = −0.41).

### Responsiveness to the intervention in terms of cut-off point, reliable change, and minimal clinically important difference criteria

At post-intervention, both for the PHQ-9 and the GAD-7, significantly lower rates of clinical cases were observed in the S-AII group compared to the N-AII and the control group (Table 4). Similarly, RCI and MCID indexed significantly higher rates of reliable and clinically important changes in the S-AII group on the PHQ-9, GAD-7, and MHC-SF scores post-intervention. But, at 1-month follow-up, no significant differences between groups were observed (see online Supplementary Table S3).

**Table 4.** Prevalence of depression and anxiety, minimal clinically important difference (MCID) and reliable change (RCI) proportions, and estimated group differences at post-intervention (*N* = 152)

	Control (*n* = 54)	N-AII (*n* = 48)	S-AII (*n* = 50)	Test statistics
Prevalence of depression (PHQ-9 ⩾ 10), *n* (%)	25 (46.3%)	26 (54.2%)	12 (24.0%)	χ^2^(2, *N* = 152) = 10.00, *p* = 0.007
Prevalence of anxiety (GAD-7 ⩾ 10), *n* (%)	26 (48.1%)	18 (37.5%)	9 (18.0%)	χ^2^(2, *N* = 152) = 10.60, *p* = 0.005
MCID on the PHQ-9				χ^2^(4, *N* = 152) = 11.05, *p* = 0.026
No change, *n* (%)	45 (83.3%)	39 (81.3%)	31 (62.0%)	
Improvement, *n* (%)	5 (9.3%)	5 (10.4%)	16 (32.0%)	
Deterioration, *n* (%)	4 (7.4%)	4 (8.3%)	3 (6.0%)	
MCID on the GAD-7				χ^2^(4, *N* = 152) = 15.47, *p* = 0.004
No change, *n* (%)	42 (77.8%)	32 (66.6%)	23 (46.0%)	
Improvement, *n* (%)	6 (11.1%)	8 (16.7%)	20 (40.0%)	
Deterioration, *n* (%)	6 (11.1%)	8 (16.7%)	7 (14.0%)	
MCID on the MHC-SF				χ^2^(4, *N* = 152) = 15.26, *p* = 0.004
No change, *n* (%)	37 (68.5%)	33 (68.7%)	23 (46.0%)	
Improvement, *n* (%)	8 (14.8%)	6 (12.5%)	21 (42.0%)	
Deterioration, *n* (%)	9 (16.7%)	9 (18.8%)	6 (12.0%)	
RCI on the PHQ-9				χ^2^(4, *N* = 152) = 11.13, *p* = 0.025
No change, *n* (%)	47 (87.0%)	42 (87.4%)	37 (74.0%)	
Improvement, *n* (%)	3 (5.6%)	3 (6.3%)	12 (24.0%)	
Deterioration, *n* (%)	4 (7.4%)	3 (6.3%)	1 (2.0%)	
RCI on the GAD-7				χ^2^(4, *N* = 152) = 13.29, *p* = 0.010
No change, *n* (%)	46 (85.2%)	36 (75.0%)	29 (58.0%)	
Improvement, *n* (%)	4 (7.4%)	7 (14.6%)	17 (34.0%)	
Deterioration, *n* (%)	4 (7.4%)	5 (10.4%)	4 (8.0%)	
RCI on the MHC-SF				χ^2^(4, *N* = 152) = 10.92, *p* = 0.027
No change, *n* (%)	41 (75.9%)	41 (85.4%)	31 (62.0%)	
Improvement, *n* (%)	6 (11.1%)	3 (6.3%)	14 (28.0%)	
Deterioration, *n* (%)	7 (13.0%)	4 (8.3%)	5 (10.0%)	

GAD-7, seven-item Generalized Anxiety Disorder Scale; MHC-SF, Mental Health Continuum-Short Form; PHQ-9, nine-item Patient Health Questionnaire; N-AII, non-affirming implementation intention condition; S-AII, self-affirming implementation intention condition. Results are based on complete case analysis.

## Discussion

The results of this study indicate positive short-term effects (i.e. over 2 weeks) on a range of mental health indices, including depression, anxiety, and well-being for adults with psoriasis, subsequent to II-based self-affirmation intervention. These findings are especially notable given that the intervention was brief, low in intensity, and the intervention effects were not augmented by the inclusion of any booster component. It is also notable that the intervention effects were compared with both the passive and active comparison groups, providing more robust evidence for the utility of S-AII.

Shedding more light on the effectiveness, this low-intensity S-AII intervention yielded small to medium effect sizes post-intervention on well-being (*d*s: 0.25–0.41), anxiety (*d*s: −0.45 to −0.60), and depression (*d*s: −0.40 to −0.55) in a clinically important sample. At first sight, these effect sizes seem not to be considerable. The observed effects, however, compare (more) favorably to the results of three meta-analyses on the effects of positive psychology interventions (PPIs; see Bolier et al., 2013; Sin & Lyubomirsky, 2009; White, Uttl, & Holder, 2019). The mean estimated effect sizes of PPIs were 0.34 on subjective well-being, 0.20 on psychological well-being, and 0.23 on depression (Bolier et al., 2013), or even smaller effects have been reported (c.f. White et al., 2019). As guided self-help with low cost and potentially substantial public health reach, the S-AII exerted thus quite considerable effects. Also, it needs to be recognized that though these brief positive single activities such as S-AII are limited in scope (being in stark contrast to comprehensive multi-component interventions), the development of brief techniques to increase well-being and mental health is invaluable in serving to identify the most beneficial activities (that may be further tested and incorporated in comprehensive interventions).

This is the first study to document short- and longer-term follow-up S-AII effects on psoriasis patients' mental health outcomes. Given that mental health and well-being deficits represent important treatment targets in adults with psoriasis and a paucity of empirical literature on the effectiveness of psychological strategies and interventions that can attenuate the adverse effects of psoriasis in a manner that builds psychological capacity, the current findings offer a valid contribution. Though the post-intervention significant differences between groups faded at 1 month after, within the S-AII group changes on all mental health outcomes over time, from baseline to 1-month follow-up assessments, remained significant. The observed effect sizes for changes in primary outcomes from baseline to follow-up ranged from 0.27 for well-being to −0.49 for anxiety. Interestingly, the results showed also some parallel improvements over time in depression and anxiety within the N-AII group that used distraction strategies (*d*s: −0.22 to −0.44). In line with previously reported findings on social anxiety, it seems that for reducing negative mental health outcomes via IIs, self-affirming content is not necessary (c.f. Łakuta, 2020a). But, remarkably, only the S-AII exerted fast and meaningful improvement and provided significant net benefits in terms of increased well-being, including its social dimension. These findings thus support its status as a well-being intervention (c.f. Howell, 2017). The results are also in line with prior correlational research showing that adults with psoriasis reporting stronger tendencies to spontaneously self-affirm in response to threats also reported having better mental health outcomes, including higher happiness and satisfaction with life, as well as lower depression and anxiety (Łakuta, 2020b). Overall, II-based self-affirmation intervention, by defocusing and promoting a broader perspective of an individual to the psychological resources residing within the self (Critcher & Dunning, 2015), can be seen as a strategy capable to favor adaptive coping, offering a potential to attenuate or halt the progression of mental health problems.

Several limitations should be acknowledged. While great efforts were taken to recruit a broad psoriasis patient sample, the participants still represented a segment of the community population that was interested and engaged to actively respond to the study, limiting the generalizability of the results. The resulting sample was not gender-balanced. There was no recruited sample with clinically diagnosed depression and/or anxiety, so conclusions are limited to a sub-clinical sample of participants being at risk/with elevated symptoms of depression and anxiety^3^. Moreover, because the observed effects are based on self-report measures, it is important for future studies to use also alternate measurement strategies. It should also be recognized that in RCT-based research, follow-up time-points are relatively arbitrary (i.e. commonly used 1-, 6-, and 12-month follow-up time-points) and this might bring forth erroneous conclusions on the effectiveness of interventions (e.g. Moore, Depp, Wetherell, & Lenze, 2016; Schuster et al., 2020). Compared to traditional assessments methods, measuring intervention effects by means of an ecological momentary assessment (EMA) approach would further help optimizing sensitivity in RCT-based research on S-AII and enhance our understanding of how S-AII intervention effectiveness develops over time. Therefore, future research on S-AII is encouraged to apply EMA when assessing outcome measures and intervention effectiveness.

The considerable improvements in mental health outcomes among participants in the S-AII condition are very encouraging. On the other hand, it is also important to note that based on MCID and RCI indexes, the S-AII intervention resulted in no major change in approximately 46% to even 74% of participants and a deterioration in primary outcomes among approximately 2–14% of participants. These findings suggest some important factors (e.g. personal, contextual, and/or processual) might not be captured in the S-AII intervention, resulting in limited effectiveness in some individuals. Future studies could consider more direct manipulation of the contextual factors conceptualized within the Trigger and Channel framework (c.f. Ferrer & Cohen, 2019) and also the role of precisely *v.* broadly specified situations defined by the ‘*if*’ component in S-AII to help advance an understanding of when self-affirmation is likely to be most effective. For example, a promising direction for future research is testing potential modifications of the S-AII aiming to further strengthen the element of specificity of the intervention (i.e. directly to body- or disease-related issues). Future studies could also seek to determine the extent that S-AII develops/strengthens the tendency to spontaneously call to mind self-affirming contents, as it may mediate affirmation effects (c.f. Brady et al., 2016). Finally, it is also worth noting that in part the data were collected during the COVID-19 outbreak that could possibly affect the results.

Despite these limitations, this study has noteworthy strengths in that it tested the target intervention outside the laboratory, in an at-risk sample (being highly-stigmatized with elevated depression, anxiety, and lowered well-being), in individuals' own settings, that was compared against active control group matched to the target condition. This study's strengths include also pre-registration, sufficient statistical power to detect medium effects assumed based on prior research, and analyses under the ITT principle (that minimizes effect overestimations), supporting its internal validity. Though significant differences between groups diminished 1 month after, and also some limited effects were observed on secondary outcomes, with the exception of the significant decrease of catastrophizing, the S-AII shows promising results as a relevant public mental health strategy for enhancing well-being and reducing anxiety and depressive symptoms. Future studies could consider whether these effects can be further enhanced with booster interventions.

## Footnotes

† The notes appear after the main text.

## Notes

1 Cohen and Sherman (2014) argue that self-affirmation affords a route toward cycles of adaptive potential, including processes of: (1) recursion, in which problem resolution further affirms the self which, in turn, promotes subsequent goal attainment; (2) interaction processes, in which others' positive reactions to one's self-affirmed identity serves to further reinforce that identity; and (3) subjective construal processes, in which future threats are interpreted in a more balanced manner given one's strengthened self-identity.

2 Between-group standardized effect sizes (Cohen's *d*) were computed by dividing the unstandardized effects (estimated mean differences between conditions) by the pre-treatment pooled standard deviation. Standardized within-group effect sizes (Cohen's *d*) were calculated by dividing the unstandardized effects (estimated mean differences) by the pre-treatment standard deviation.

3 Introduced into the study only people who are experiencing at least moderate depressive and anxiety symptoms would also result in selection bias limiting the generalizability of the findings. Exclusionary practices as insufficient or too severe symptoms exclude a large proportion of a representative cohort of individuals from trial participation limiting the generalizability of the findings, reducing the confidence that findings can be translated into real-world settings, and resulting in reported overestimated effects. The generalizability could be thus limited due to exclusion based on the use of minimum baseline severity.

## Supplementary material

For supplementary material accompanying this paper visit https://doi.org/10.1017/S0033291721004499. click here to view supplementary material
