# Supplementary material for: Brief self-affirmation intervention for adults with psoriasis for reducing anxiety and depression and boosting well-being: Evidence from a randomized controlled trial
Source: Psychol Med. 2021 Nov 5;53(6):2574–84. doi: 10.1017/S0033291721004499 (PMC10123834; doi:10.1017/S0033291721004499)
Supplement: Supplementary file 1 [file S0033291721004499sup001.docx]

**Supplement**

**Brief self-affirmation intervention for adults with psoriasis for reducing anxiety and depression and boosting well-being: Evidence from a randomized controlled trial**

**Confirmatory hypotheses**

Eight pre-registered hypotheses were tested in the study. It was hypothesized that participants in the S-AII intervention condition would experience significantly lower depression (H1) and anxiety (H2) levels and higher well-being (H3) relative to participants in the N-AII and control condition (a) two weeks post-intervention and (b) at the follow-up, one month later. Turning to the secondary outcomes, it was hypothesized that participants in the S-AII intervention condition would experience significantly higher levels of adaptive cognitive emotion regulation strategies, including acceptance, refocusing on planning, positive reappraisal, positive refocusing, and putting into perspective (H4); lower levels of maladaptive cognitive emotion regulation strategies, including self-blame, rumination, catastrophizing, and other-blame (H5); higher levels of positive other-directed feelings (H6); higher levels of positive self-directed feelings (H7); and, lower levels of negative emotional attitude towards the body (H8) relative to participants in the N-AII and control condition (a) two weeks post-intervention and (b) at the follow-up one month later.

**Reliable Change and Minimal Clinically Important Difference Indices**

A reliable change in sum scores was established on the basis of the reliable change index (RCI; Jacobson & Truax, 1991). RCI identifies a level of change between two time-points of assessment that is required to classify, with 95% confidence, that the detected difference is reliable and real beyond what can be attributed to measurement error. If the difference in scores between two time-points of assessment for a person exceeds this level, the person can be classified as making reliable change. The RCI can be used to identify people showing a reliable improvement or a reliable deterioration in scores. In the present study, align with the formula defined by Jacobson and Truax (1991), the RCI for the PHQ-9, GAD-7, and MHC-SF was 5.74, 4.55, and 9.59, respectively. These results are in line with results from previous studies (e.g., Smith et al., 2017; Jones et al., 2019). Consequently, within-person changes, up or down, greater than those values were regarded as reliable.

Furthermore, the minimal clinically important differences (MCIDs) in the primary outcome scores were determined. The MCID is the smallest change in a treatment outcome that can be considered to be worthwhile or clinically important (i.e., the level of change an individual patient would identify as important, and which would indicate a change in the patient's management; Cook, 2008; Mouelhi et al., 2020; Wright et al., 2012). It is a context-specific value. To estimate the intraindividual MCID a standard error of measurement (SEM) is used (c.f., Wright et al., 2012; Wyrwich, Tierney, & Wolinsky, 1999). Using either a 1-SEM change criterion or a 2-SEM change criterion (a more conservative estimate) is typically a suitable method for evaluating clinically relevant change in individual patients (Kroenke et al., 2016, 2018; Löwe et al., 2004; Wright et al., 2012). In the current study, to establish intraindividual MCIDs that reflect the 95% confidence interval, the estimated SEMs for the PHQ-9, GAD-7, and MHC-SF were multiplied by 1.96, align with a more conservative approach. Accordingly, the MCIDs for the PHQ-9, GAD-7, and MHC-SF were 4.24, 3.22, and 6.78, respectively. As a result, a clinically meaningful response to the intervention was defined as achieving MCID for the PHQ-9, GAD-7, and MHC-SF equivalent to a 5-, a 4-, and a 7-point change (or greater) in total score, respectively. These estimates are in line with results from previous studies (e.g., Kroenke et al. 2019; Toussaint et al., 2020).

**Measures (Extended)**

***Primary Outcome Measures***

The three pre-registered primary outcomes captured depression and anxiety severity and well-being.

Depression severity was measured by the 9-item Patient Health Questionnaire (PHQ-9; Kroenke et al., 2001). Items are rated on a 4-point Likert-type scale from 0 (*not at all*) to 3 (*nearly every day*). It is one of the most frequently used diagnostic self-report scales for screening, diagnosis, and severity assessment of major depression (c.f., Levis et al., 2019). The PHQ-9 is well validated against standard criteria, has demonstrated sensitivity to change, and is used in a variety of clinical and non-clinical settings (e.g., Blenkiron & Goldsmith, 2019; Horton & Perry, 2016; McMillan et al., 2010). In the current research, Cronbach's alpha coefficients through assessment points ranged from 0.87 to 0.90 (*M*_⍺_ = 0.88).

Anxiety severity was measured by the 7-item Generalized Anxiety Disorder Scale (GAD-7; Spitzer et al., 2006). Items are rated on a 4-point Likert-type scale from 0 (*not at all*) to 3 (*nearly every day*). It is one of the most frequently used diagnostic self-report scales for screening, diagnosis, and severity assessment of generalized anxiety disorder, moreover, the scale is used as a measure for anxiety in general and in anxiety disorder research (c.f., Johnson et al., 2019; Löwe et al., 2008). The GAD-7 is well validated against standard criteria, has demonstrated sensitivity to change, and is used in a variety of clinical and non-clinical settings (e.g., Blenkiron & Goldsmith, 2019; Johnson et al., 2019). In the current research, Cronbach's alpha coefficients through assessment points ranged from 0.90 to 0.92 (*M*_⍺_ = 0.91).

Well-being was measured with the 14-item Mental Health Continuum–Short Form (MHC-SF; Karaś, Cieciuch, & Keyes, 2014; Keyes et al., 2002; Lamers et al., 2011). The MHC-SF measures three dimensions of well-being: (1) hedonic, emotional well-being (three items), which relates to positive emotions and life satisfaction; (2) eudaimonic, social well-being (five items), which relates to one’s functioning in society (i.e., social contribution, social integration, social actualization/growth, social acceptance, social coherence/interest); and (3) eudaimonic, psychological well-being (six items), which relates to optimal individual functioning (i.e., self acceptance, environmental mastery, positive relations with others, personal growth, autonomy, and purpose in life). Items are rated on a 6-point scale ranging from 0 (*never*) to 5 (*every day*). In this study, the total scale scores, as well as the subscale scores, were used. The MHC-SF is well validated, has demonstrated sensitivity to change, and is used in a variety of clinical and non-clinical settings (e.g., Ferentinos et al., 2019; Weiss et al., 2016). In the current research, Cronbach's alpha coefficients for total scale scores through assessment points ranged from 0.94 to 0.96 (*M*_⍺_ = 0.95). For subscale scores, Cronbach's alpha coefficients through assessment points ranged from 0.87 to 0.92 (*M*_⍺_ = 0.90).

***Secondary Outcome Measures***

Besides primary outcomes, pre-registered additional effects of the intervention in terms of cognitive emotion regulation strategies, positive other-directed and self-directed feelings, and emotional attitude toward the body.

Cognitive emotion regulation strategies were measured by the Cognitive Emotion Regulation Questionnaire (Garnefski et al., 2002; Marszał-Wiśniewska & Fajkowska, 2010). It consists of 36 items in 9 subscales, including four maladaptive (i.e., self-blame, rumination, catastrophizing, and other-blame) and five adaptive (i.e., acceptance, refocusing on planning, positive reappraisal, positive refocusing, and putting into perspective) emotion regulation strategies. Each subscale includes four items rated from 1 (*almost never*) to 5 (*almost always*). The CERQ has been used in multiple populations, in both general and clinical samples (e.g., Feliu-Soler et al., 2017; Garnefski & Kraaij, 2006; Li et al., 2019), showing adequate reliability and validity. In the current research, Cronbach's alpha coefficients through assessment points ranged from 0.60 (for acceptance scale) to 0.92 (other-blame scale) with *M*_⍺_ = 0.83.

Positive other-directed feelings were measured by asking participants to indicate how often they have experienced five positive prosocial feelings (e.g., love, empathic, connected, grateful) in their daily lives (Crocker et al., 2008; Thomaes et al., 2012). Items are rated on a 5-point-scale ranged from 0 (*very rarely or never*) to 4 (*very often or always*). In the current research, Cronbach's alpha coefficients through assessment points ranged from 0.82 to 0.89 (*M*_⍺_ = 0.86).

Positive self-directed feelings were measured by asking participants to indicate how often they have experienced five positive feelings directed toward themselves (e.g., pride, feeling strong, in control) in their daily lives (Crocker et al., 2008; Thomaes et al., 2012). Items are rated on a 5-point-scale ranged from 0 (*very rarely or never*) to 4 (*very often or always*). In the current research, Cronbach's alpha coefficients through assessment points ranged from 0.86 to 0.90 (*M*_⍺_ = 0.88).

Negative emotional attitude towards the body was measured by the 9-item Body Emotions Scale derived from the Body Self Questionnaire (Sakson-Obada, 2009). Items are rated on a 7-point Likert-type scale (sample items: “I am ashamed of how I look”, “Sometimes I hate the way I look”), ranging from 1 (*strongly disagree*) to 7 (*strongly agree*). The scale has demonstrated good reliability and validity (e.g., Sakson-Obada, 2009; Łakuta et al., 2016). In the current research, Cronbach's alpha coefficients through assessment points ranged from 0.90 to 0.93 (*M*_⍺_ = 0.92).

**Table S1.** Descriptive statistics of secondary outcomes at baseline by the study arms (*N* = 175)

|  | | Condition | | *M* | | *SD* | |
| --- | --- | --- | --- | --- | --- | --- | --- |
| Positive self-directed feelings |  | Control |  | 8.78 |  | 4.21 |  |
|  |  | N-AII |  | 8.10 |  | 4.26 |  |
|  |  | S-AII |  | 8.74 |  | 4.13 |  |
| Positive other-directed feelings |  | Control |  | 12.53 |  | 4.49 |  |
|  |  | N-AII |  | 11.83 |  | 4.55 |  |
|  |  | S-AII |  | 11.83 |  | 3.97 |  |
| Negative emotional attitude towards the body |  | Control |  | 47.37 |  | 10.65 |  |
|  |  | N-AII |  | 48.88 |  | 9.19 |  |
|  |  | S-AII |  | 46.55 |  | 10.69 |  |
| Self-blame |  | Control |  | 11.58 |  | 3.34 |  |
|  |  | N-AII |  | 11.50 |  | 3.70 |  |
|  |  | S-AII |  | 11.10 |  | 3.24 |  |
| Acceptance |  | Control |  | 13.27 |  | 2.29 |  |
|  |  | N-AII |  | 13.09 |  | 2.66 |  |
|  |  | S-AII |  | 13.03 |  | 2.78 |  |
| Rumination |  | Control |  | 12.81 |  | 3.80 |  |
|  |  | N-AII |  | 12.72 |  | 3.94 |  |
|  |  | S-AII |  | 12.69 |  | 3.77 |  |
| Positive refocusing |  | Control |  | 11.10 |  | 2.90 |  |
|  |  | N-AII |  | 10.64 |  | 3.65 |  |
|  |  | S-AII |  | 11.64 |  | 3.85 |  |
| Refocusing on planning |  | Control |  | 14.23 |  | 2.66 |  |
|  |  | N-AII |  | 13.71 |  | 3.09 |  |
|  |  | S-AII |  | 14.55 |  | 3.58 |  |
| Positive reappraisal |  | Control |  | 11.93 |  | 3.16 |  |
|  |  | N-AII |  | 11.41 |  | 3.67 |  |
|  |  | S-AII |  | 12.38 |  | 3.75 |  |
| Putting into perspective |  | Control |  | 13.10 |  | 3.02 |  |
|  |  | N-AII |  | 12.10 |  | 3.09 |  |
|  |  | S-AII |  | 12.14 |  | 3.32 |  |
| Catastrophizing |  | Control |  | 10.34 |  | 3.73 |  |
|  |  | N-AII |  | 10.93 |  | 3.20 |  |
|  |  | S-AII |  | 10.16 |  | 3.11 |  |
| Other-blame |  | Control |  | 9.83 |  | 3.68 |  |
|  |  | N-AII |  | 9.76 |  | 3.60 |  |
|  |  | S-AII |  | 9.41 |  | 3.30 |  |

*Note*. N-AII = non-affirming implementation intention condition; S-AII = self-affirming implementation intention condition.

**Table S2.** Estimated means, 95% confidence intervals for mean changes, and within-group effect sizes (*N* = 175)

| Outcome | Condition | Estimated mean (S.E.) | Time | Estimated mean difference (95% CI) | *p* | Cohen’s *d* |
| --- | --- | --- | --- | --- | --- | --- |
| PHQ-9 | Control | T1: 9.83 (0.80) | T2 vs. T1 | 0.20 (-0.99; 1.39) | .737 | 0.03 |
|  |  | T2: 10.03 (0.79) | T3 vs. T1 | -0.47 (-2.18; 1.24) | .589 | -0.08 |
|  |  | T3: 9.36 (0.86) | T3 vs. T2 | -0.67 (-2.05; 0.71) | .337 | -0.11 |
|  | N-AII | T1: 11.40 (0.81) | T2 vs. T1 | -0.29 (-1.54; 0.96) | .650 | -0.04 |
|  |  | T2: 11.11 (0.81) | T3 vs. T1 | -1.80 (-3.54; -0.65) | **.042** | -0.28 |
|  |  | T3: 9.60 (0.88) | T3 vs. T2 | -1.51 (-2.90; -0.13) | **.032** | -0.23 |
|  | S-AII | T1: 10.28 (0.81) | T2 vs. T1 | -2.65 (-3.88; -1.42) | **< .001** | -0.44 |
|  |  | T2: 7.62 (0.80) | T3 vs. T1 | -2.15 (-3.87; -0.44) | **.014** | -0.35 |
|  |  | T3: 8.12 (0.87) | T3 vs. T2 | 0.50 (-0.87; 1.86) | .437 | 0.08 |
| GAD-7 | Control | T1: 9.59 (0.68) | T2 vs. T1 | -0.04 (-1.16; 1.09) | .948 | -0.01 |
|  |  | T2: 9.56 (0.65) | T3 vs. T1 | -1.43 (-3.09; 0.22) | .089 | -0.27 |
|  |  | T3: 8.16 (0.79) | T3 vs. T2 | -1.40 (-2.75; -0.04) | **.043** | -0.27 |
|  | N-AII | T1: 10.02 (0.68) | T2 vs. T1 | -1.19 (-2.37; -0.02) | **.047** | -0.22 |
|  |  | T2: 8.82 (0.67) | T3 vs. T1 | -2.36 (-4.03; -0.69) | **.006** | -0.44 |
|  |  | T3: 7.66 (0.80) | T3 vs. T2 | -1.17 (-2.52; 0.19) | .092 | -0.22 |
|  | S-AII | T1: 9.22 (0.68) | T2 vs. T1 | -2.72 (-3.88; -1.56) | **< .001** | -0.55 |
|  |  | T2: 6.50 (0.66) | T3 vs. T1 | -2.46 (-4.11; -0.80) | **.004** | -0.49 |
|  |  | T3: 6.77 (0.79) | T3 vs. T2 | 0.27 (-1.08; 1.61) | .697 | 0.05 |
| MHC-SF  (total score) | Control | T1: 27.31 (2.02) | T2 vs. T1 | -1.07 (-3.40; 1.26) | .368 | -0.07 |
|  |  | T2: 26.24 (1.97) | T3 vs. T1 | 1.31 (-2.46; 5.07) | .494 | 0.08 |
|  |  | T3: 28.61 (2.38) | T3 vs. T2 | 2.38 (-0.64; 5.39) | .122 | 0.15 |
|  | N-AII | T1: 24.45 (2.03) | T2 vs. T1 | -0.79 (-3.25; 1.67) | .526 | -0.05 |
|  |  | T2: 23.66 (2.02) | T3 vs. T1 | 1.04 (-2.79; 4.88) | .592 | 0.06 |
|  |  | T3: 25.49 (2.42) | T3 vs. T2 | 1.84 (-1.18; 4.85) | .231 | 0.11 |
|  | S-AII | T1: 26.40 (2.03) | T2 vs. T1 | 3.65 (1.24; 6.06) | **.003** | 0.25 |
|  |  | T2: 30.05 (2.00) | T3 vs. T1 | 4.01 (0.23; 7.78) | **.038** | 0.27 |
|  |  | T3: 30.40 (2.40) | T3 vs. T2 | 0.36 (-2.62; 3.33) | .813 | 0.02 |

*Note*. GAD-7 = 7-item Generalized Anxiety Disorder Scale; MHC-SF = Mental Health Continuum–Short Form; PHQ-9 = 9-item Patient Health Questionnaire; S.E. = standard error; T1 = baseline assessment; T2 = week 2 (post-intervention) assessment; T3 = 1-month follow-up assessment. Bolded values denote statistical significance at the *p* < .05 level.

**Table S3.** Prevalence of depression and anxiety, minimal clinically important difference (MCID) and reliable change (RCI) proportions and group differences at 1-month follow-up (*N* = 109)

|  | Control  (*n* = 36) | N-AII  (*n* = 36) | S-AII  (*n* = 37) | Test Statistics |
| --- | --- | --- | --- | --- |
| Prevalence of depression (PHQ-9 ≥ 10), *n* (%) | 15 (41.7%) | 13 (36.1%) | 15 (40.5%) | χ^2^(2, *N* = 109) = 0.26, *p* = .878 |
| Prevalence of anxiety (GAD-7 ≥ 10), *n* (%) | 13 (36.1%) | 7 (19.4%) | 10 (27.0%) | χ^2^(2, *N* = 109) = 2.51, *p* = .285 |
| MCID on the PHQ-9 |  |  |  | χ^2^(4, *N* = 109) =1.42, *p* = .840 |
| no change, *n* (%) | 26 (72.2%) | 25 (69.4%) | 25 (67.6%) |  |
| improvement, *n* (%) | 5 (13.9%) | 8 (22.2%) | 8 (21.6%) |  |
| deterioration, *n* (%) | 5 (13.9%) | 3 (8.3%) | 4 (10.8%) |  |
| MCID on the GAD-7 |  |  |  | χ^2^(4, *N* = 109) = 1.16, *p* = .885 |
| no change, *n* (%) | 20 (55.6%) | 22 (61.1%) | 21 (56.8%) |  |
| improvement, *n* (%) | 9 (25.0%) | 10 (27.8%) | 11 (29.7%) |  |
| deterioration, *n* (%) | 7 (19.4%) | 4 (11.1%) | 5 (13.5%) |  |
| MCID on the MHC-SF |  |  |  | χ^2^(4, *N* = 109) = 1.94, *p* = .747 |
| no change, *n* (%) | 17 (47.2%) | 19 (52.8%) | 17 (46.0%) |  |
| improvement, *n* (%) | 8 (22.2%) | 10 (27.8%) | 12 (32.4%) |  |
| deterioration, *n* (%) | 11 (30.6%) | 7 (19.4%) | 8 (21.6%) |  |
| RCI on the PHQ-9 |  |  |  | χ^2^(4, *N* = 109) = 4.66, *p* = .324 |
| no change, *n* (%) | 29 (80.6%) | 28 (77.8%) | 26 (70.3%) |  |
| improvement, *n* (%) | 3 (8.3%) | 7 (19.4%) | 7 (18.9%) |  |
| deterioration, *n* (%) | 4 (11.1%) | 1 (2.8%) | 4 (10.8%) |  |
| RCI on the GAD-7 |  |  |  | χ^2^(4, *N* = 109) = 2.57, *p* = .633 |
| no change, *n* (%) | 25 (69.4%) | 24 (66.7%) | 24 (64.9%) |  |
| improvement, *n* (%) | 5 (13.9%) | 9 (25.0%) | 9 (24.3%) |  |
| deterioration, *n* (%) | 6 (16.7%) | 3 (8.3%) | 4 (10.8%) |  |
| RCI on the MHC-SF |  |  |  | χ^2^(4, *N* = 109) = 1.47, *p* = .833 |
| no change, *n* (%) | 20 (55.6%) | 23 (63.9%) | 22 (59.5%) |  |
| improvement, *n* (%) | 7 (19.4%) | 6 (16.7%) | 9 (24.3%) |  |
| deterioration, *n* (%) | 9 (25.0%) | 7 (19.4%) | 6 (16.2%) |  |

*Note*. GAD-7 = 7-item Generalized Anxiety Disorder Scale; MHC-SF = Mental Health Continuum–Short Form; PHQ-9 = 9-item Patient Health Questionnaire; N-AII = non-affirming implementation intention condition; S-AII = self-affirming implementation intention condition. Analyses of observed data (complete case analyses).

**References**

Blenkiron, P., & Goldsmith, L. (2019). Patient-reported outcome measures in community mental health teams: Pragmatic evaluation of PHQ-9, GAD-7 and SWEMWBS. *BJPsych Bulletin, 43*, 221-227. doi: 10.1192/bjb.2019.20

Cook C. E. (2008). Clinimetrics Corner: The Minimal Clinically Important Change Score
(MCID): A Necessary Pretense. *The Journal of Manual & Manipulative Therapy, 16*(4), E82–E83. doi: 10.1179/jmt.2008.16.4.82E

Crocker, J., Niiya, Y., & Mischkowski, D. (2008). Why does writing about important values reduce defensiveness? *Psychological Science, 19*, 740-747. doi: 10.1111/j.1467-9280.2008.02150.x

Feliu-Soler, A., Reche-Camba, E., Borràs, X., Pérez-Aranda, A., Andrés-Rodríguez, L., Peñarrubia-María, M. T., … Luciano, J. V. (2017). Psychometric properties of the Cognitive Emotion Regulation Questionnaire (CERQ) in patients with fibromyalgia syndrome. *Frontiers in Psychology, 8*, 2075. doi: 10.3389/fpsyg.2017.02075

Ferentinos, P., Yotsidi, V., Porichi, E., Douzenis, A., Papageorgiou, C., & Stalikas, A. (2019). Well‐being in patients with affective disorders compared to nonclinical participants: A multi‐model evaluation of the Mental Health Continuum‐Short Form. *Journal of Clinical Psychology, 75*, 1585-1612. doi: 10.1002/jclp.22780

Garnefski, N., & Kraaij, V. (2006). Relationships between cognitive emotion regulation strategies and depressive symptoms: A comparative study of five specific samples. *Personality and Individual Differences, 40*, 1659-1669. doi: 10.1016/j.paid.2005.12.009

Garnefski, N., Kraaij, V., & Spinhoven, Ph. (2002). *CERQ: Manual for the use of the Cognitive Emotion Regulation Questionnaire. A questionnaire for measuring cognitive coping strategies*. Leiderdorp: DATEC V.O.F.

Horton, M., & Perry, A. E. (2016). Screening for depression in primary care: A Rasch analysis of the PHQ-9. *BJPsych Bulletin, 40*, 237-243. doi: 10.1192/pb.bp.114.050294

Jacobson, N. S., & Truax, P. (1991). Clinical significance: A statistical approach to defining meaningful change in psychotherapy research. *Journal of Consulting and Clinical Psychology, 59*(1), 12–19. doi: 10.1037/ 0022-006X.59.1.12

Jones, S. M. W., Crane, P. K., & Simon, G. (2019). A comparison of individual change using Item Response Theory and sum scoring on the Patient Health Questionnaire-9. *Annals of Depression and Anxiety, 6*(1), 1098. doi: 10.26420/anndepressanxiety.1098.2019

Karaś, D., Cieciuch, J., & Keyes, C. L. M. (2014). The Polish Adaptation of the Mental Health Continuum-Short Form (MHC-SF). *Personality and Individual Differences, 69*, 104-109. doi: 10.1016/j.paid.2014.05.011

Keyes, C. L. M. (2002). The mental health continuum: From languishing to flourishing in life. *Journal of Health and Social Behavior, 43*, 207-222. doi: 10.2307/3090197

Kroenke, K., Baye, F., & Lourens, S. G. (2019). Comparative validity and responsiveness of PHQ-ADS and other composite anxiety-depression measures. *Journal of Affective Disorders, 246*, 437–443. doi: 10.1016/j.jad.2018.12.098

Kroenke, K., Spitzer, R. L., & Williams, J. B. (2001). The PHQ-9: validity of a brief depression severity measure. *Journal of General Internal Medicine, 16*(9), 606–613. doi: 10.1046/j.1525-1497.2001.016009606.x

Kroenke, K., Wu, J., Yu, Z., Bair, M. J., Kean, J., Stump, T., & Monahan, P. O. (2016). Patient Health Questionnaire Anxiety and Depression Scale: Initial Validation in Three Clinical Trials. *Psychosomatic Medicine, 78*(6), 716–727. doi: 10.1097/PSY.0000000000000322

Lamers, S. M. A., Westerhof, G. J., Bohlmeijer, E. T., ten Klooster, P. M., & Keyes, C. L. (2011). Evaluating the psychometric properties of the Mental Health Continuum-Short Form (MHC-SF). *Journal of Clinical Psychology, 67*, 99 –110. doi: 10.1002/jclp.20741

Levis, B., Benedetti, A., Thombs, B. D., & DEPRESsion Screening Data (DEPRESSD) Collaboration (2019). Accuracy of Patient Health Questionnaire-9 (PHQ-9) for screening to detect major depression: individual participant data meta-analysis. *BMJ (Clinical research ed.), 365*, l1476. http://doi.org/10.1136/bmj.l1476

Li, L., Li, S., Wang, Y., Yang, Y., & Zhu, X. (2019). Factor structure and measurement invariance for the Cognitive Emotion Regulation Questionnaire (CERQ) among women newly diagnosed with breast cancer. *Frontiers in Psychology, 10*, 1132. doi: 10.3389/fpsyg.2019.01132

Löwe, B., Decker, O., Müller, S., Brähler, E., Schellberg, D., Herzog, W., & Herzberg, P.Y. (2008). Validation and standardization of the Generalized Anxiety Disorder Screener (GAD-7) in the general population. *Medical Care, 46*, 266-274. doi: 10.1097/MLR.0b013e318160d093

Löwe, B., Unützer, J., Callahan, C. M., Perkins, A. J., & Kroenke, K. (2004). Monitoring depression treatment outcomes with the patient health questionnaire-9. *Medical Care, 42*(12), 1194–1201. doi: 10.1097/00005650-200412000-00006

Łakuta, P., Marcinkiewicz, K., Bergler-Czop, B., & Brzezińska-Wcisło, L. (2016). The relationship between psoriasis and depression: A multiple mediation model. *Body Image, 19*, 126-132. doi: 10.1016/j.bodyim.2016.08.004

Marszał-Wiśniewska, M., & Fajkowska, M. (2010). Właściwości psychometryczne Kwestionariusza Poznawczej Regulacji Emocji (CERQ) – wyniki badań na polskiej próbie [Psychometic properties of the Cognitive Emotion Regulation Questionnaire (CERQ): Results of the studies on the Polish sample]. *Studia Psychologiczne, 48*, 19-39.

McMillan, D., Gilbody, S., & Richards, D. (2010). Defining successful treatment outcome in depression using the PHQ-9: A comparison of methods. *Journal of Affective Disorders, 127*, 122-129. doi: 10.1016/j.jad.2010.04.030

Mouelhi, Y., Jouve, E., Castelli, C., & Gentile, S. (2020). How is the minimal clinically important difference established in health-related quality of life instruments? Review of anchors and methods. *Health and Quality of Life Outcomes, 18*(1), 136. doi: 10.1186/s12955-020-01344-w

Sakson–Obada, O. (2009). *Pamięć ciała. Ja cielesne w relacji przywiązania i w traumie [Memory of a body. Body ego in trauma and attachment relationship]*. Warszawa: Diffin.

Smith, J., Newby, J. M., Burston, N., Murphy, M. J., Michael, S., Mackenzie, A., Kiln, F., Loughnan, S. A., O'Moore, K. A., Allard, B. J., Williams, A. D., & Andrews, G. (2017). Help from home for depression: A randomised controlled trial comparing internet-delivered cognitive behaviour therapy with bibliotherapy for depression. *Internet Interventions, 9*, 25–37. doi: 10.1016/j.invent.2017.05.001

Spitzer, R. L., Kroenke, K., Williams, J. B. W., & Löwe, B. (2006). A brief measure for assessing generalized anxiety disorder. *Archives of Internal Medicine, 166*, 1092-1097. doi: 10.1001/archinte.166.10.1092

Thomaes, S., Bushman, B. J., de Castro, B. O., & Reijntjes, A. (2012). Arousing “gentle passions” in young adolescents: Sustained experimental effects of value affirmations on prosocial feelings and behaviors. *Developmental Psychology, 48*, 103-110. doi: 10.1037/a0025677

Toussaint, A., Hüsing, P., Gumz, A., Wingenfeld, K., Härter, M., Schramm, E., & Löwe, B. (2020). Sensitivity to change and minimal clinically important difference of the 7-item Generalized Anxiety Disorder Questionnaire (GAD-7). *Journal of Affective Disorders, 265*, 395–401. doi: 10.1016/j.jad.2020.01.032

Weiss, L. A., Westerhof, G. J., & Bohlmeijer, E. T. (2016). Can we increase psychological well-being? The effects of interventions on psychological well-being: A meta-analysis of randomized controlled trials. *PloS One, 11*(6), e0158092. doi: 10.1371/journal.pone.0158092

Wright, A., Hannon, J., Hegedus, E. J., & Kavchak, A. E. (2012). Clinimetrics corner: a closer look at the minimal clinically important difference (MCID). *The Journal of Manual & Manipulative Therapy, 20*(3), 160–166. doi: 10.1179/2042618612Y.0000000001

Wyrwich, K. W., Tierney, W. M., & Wolinsky, F. D. (1999). Further evidence supporting an SEM-based criterion for identifying meaningful intra-individual changes in health-related quality of life. *Journal of Clinical Epidemiology, 52*(9), 861–873. doi: 10.1016/s0895-4356(99)00071-2
